# Intraoperative endoprosthesis customization for repair of an aortoenteric fistula in an emergency context: a case report

**DOI:** 10.1590/1677-5449.200179

**Published:** 2021-08-02

**Authors:** Hugo Back Carrijo, Josué Rafael Ferreira Cunha, Carlos André Schuler, Marcos Aurélio Perciano Borges

**Affiliations:** 1 Hospital de Base do Distrito Federal – HBDF, Brasília, DF, Brasil.; 2 Instituto de Cardiologia do Distrito Federal – ICDF, Brasília, DF, Brasil.

**Keywords:** aortoenteric fistula, aortic aneurysm, endovascular procedures, modified endografts

## Abstract

Aortoenteric fistula is a severe clinical condition and its management remains a major technical challenge for surgeons. In these cases, the conventional surgical approach is associated with high rates of morbidity and mortality. Endovascular surgery is an excellent option in these cases, but considering that the aorta has been treated previously, anatomy may not be compatible with commercially available endovascular devices and so physician-modified endografts may be needed in urgent cases. The case reported involves a secondary aortoenteric fistula, treated on an emergency basis with endovascular techniques, using a physician-modified endograft.

## INTRODUCTION

Aortoenteric fistulae are rare complications, but they constitutes a severe threat to life. Despite the time elapsed since they were first described by Ashley Cooper in 1818,^1^ they remain a major therapeutic challenge today.

Aortoenteric fistulae are classified as primary or secondary, of which the primary type are extremely rare, caused by voluminous aneurysms that erode the adjacent enteric tract, while secondary fistulae are more common, originating in prosthetic aortic grafts, with prevalence in the range of 0.3 to 1.6% of cases.^2^ Clinical presentation may include abdominal pains, gastrointestinal hemorrhage, and a pulsating abdominal mass. However, diagnosis tends to be a challenge, since this classical triad is only seen in 23% of patients.^3^^,^^4^

Diagnosis is normally made by angiotomography, a method that offers considerable advantages over other imaging exams, since it is an easily accessible, high-resolution examination with low invasivity and a short acquisition time. Conventional surgery to correct a secondary aortoenteric fistula is traditionally based on construction of an extra-anatomic bypass, ligature of the aorta, and removal of the previous graft. However, this approach is associated with high morbidity and mortality, with operative mortality of 25 to 90% and lower limb amputation rates of 5 to 25%.^5^^,^^6^ In this context, the endovascular approach emerges as a valuable treatment option for repair of aortoenteric fistulae, considering the severity of the condition and the morbidity of conventional surgical procedures.

However, the complex anatomy encountered in many of these patients cannot be ignored, since the endoprostheses available at the time of intervention are unlikely to suit the patient’s anatomy. Some authors have described successful modifications to endoprostheses, whether involving resection of segments or construction of fenestrations. This solution is habitually reserved for emergency cases, to enable endovascular repair.^7^^-^9

The Research Ethics Committee approved this study (decision number 4.748.984).

## CASE REPORT

A 66-year-old male patient developed upper digestive hemorrhage, with two episodes of hematemesis, 2 days prior to hospital admission, and several episodes of melena thereafter. Fifteen years previously, he had undergone open surgery to perform right aortofemoral and left external iliac bypass with a Dacron graft. He also had a history of systemic arterial hypertension and chronic obstructive pulmonary disease.

**Figure 1:**
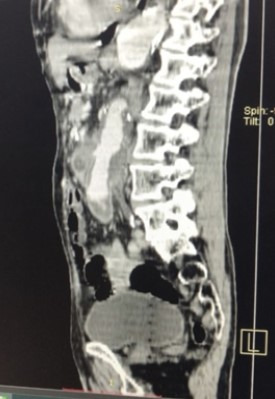
Sagittal angiotomography image showing aortoenteric fistula in the region of the proximal anastomosis of the prior aortic bypass.

**Figure 2:**
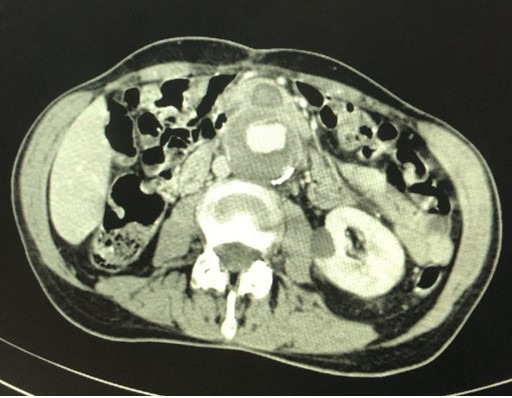
Axial angiotomography image of the abdomen showing aortoenteric fistula in the region of the proximal anastomosis of the prior aortic bypass.

Abdominal angiotomography showed saccular dilation of the infrarenal abdominal aorta proximal of the bypass, communicating with the fourth portion of the duodenum and measuring 51x41 mm, suggestive of pseudoaneurysm of the anastomosis proximal of the graft, associated with an aortoenteric fistula (Figure 1 and 2).

Since this was a secondary aortoenteric fistula, linked to a prior abdominal intervention in a patient with very poor clinical status, the decision was taken to use an endovascular approach to repair the injury. However, while using angiotomography reconstruction to take measurements to plan endovascular repair, it was found that the patient’s anatomic parameters were unsuitable, for two main reasons:

1. The proximal neck diameter was 36 mm, preventing use of bifurcated endografts. It should be remembered that the largest diameters available are of the order of 36 mm;
2. The distance between the lower renal artery and the bifurcation in the Dacron graft used for the right aortofemoral and left external iliac bypass was 89 mm, ruling out use of a thoracic aorta stent graft, since the shortest devices have a length of 100 mm.

Faced with these anatomic conditions, there was therefore no comercially available endovascular device that could be used immediately.

Given the urgency and severity of the case, combined with the impossibility of ordering a customized endovascular device, the decision was taken to perform intraoperative customization of a 40x167 mm Medtronic Valiant Captivia thoracic stent graft (Medtronic, Minnesota, United States).

**Figure 3:**
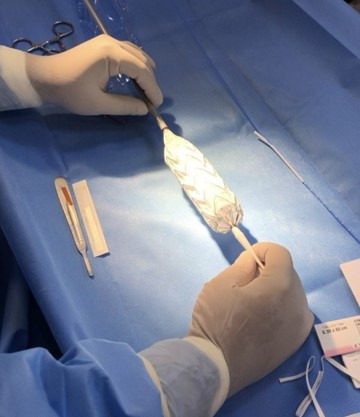
Releasing the Medtronic Valiant Captivia stent graft (Medtronic, Minnesota, United States).

**Figure 4:**
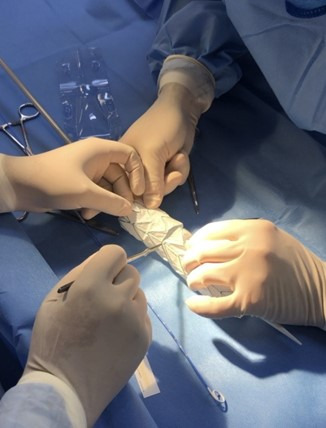
Resection of the distal segment of the Medtronic Valiant Captivia stent graft (Medtronic, Minnesota, United States).

First, the device was released on a surgical back table (Figure 3). Next, the first stage of the Medtronic Valiant Captivia stent graft was released, maintaining its open proximal ring fixed. Then measurements were taken of the device and the excess distal portion was resected with a scalpel blade (Figure 4).

**Figure 5:**
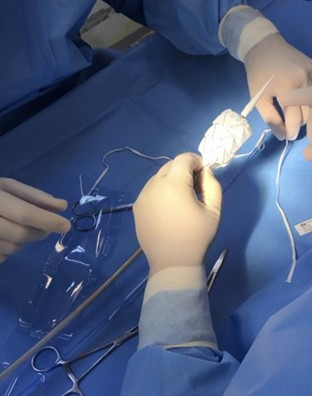
Re-sheathing the Medtronic Valiant Captivia stent graft (Medtronic, Minnesota, United States).

**Figure 6:**
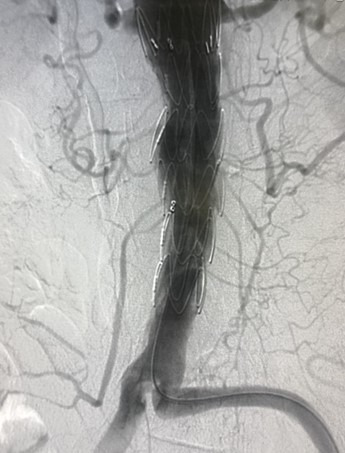
Final angiography showing complete exclusion of the pseudoaneurysm and resolution of the aortoenteric fistula.

The stent graft was then re-sheathed, with the aid of cardiac tape, slowly and progressively, until the entire length of the device was completely covered by the delivery and release system (Figure 5). For the procedure, the right femoral artery was dissected for insertion of a 7F introducer and a 5F introducer was inserted into the left femoral artery via an ultrasound-guided puncture. The stent graft was introduced via the right common femoral artery, over a Lunderquist guidewire (Cook Group Inc., Indiana, United States), and positioned infrarenally, covering the pseudoaneurysm of the proximal anastomosis of the previous aortic bypass. There were no complications during deployment of the stent graft and the final angiographic control demonstrated complete exclusion of the pseudoaneurysm and resolution of the aortoenteric fistula (Figure 6).

The patient recovered well during the postoperative period, was discharged from hospital after 72h, adapting well to oral feeding, with no intercurrent conditions, and with no further bleeding episodes.

## DISCUSSION

Aortoenteric fistulae are one of the most serious complications of aneurysms of the aorta and they tend to have high postoperative mortality rates.^10^ Open repair is associated with significant surgical morbidity, bearing in mind the technical difficulties involved in revisiting a previously operated abdomen, the risk of bleeding and of significant contamination, and the low patency of extra-anatomic revascularization.^11^

A review that analyzed 216 articles with a total of 823 patients with aortoenteric fistula demonstrated that the intra-hospital mortality associated with the endovascular approach is less than that associated with the open approach (7.1% versus 33.9%, respectively), with no statistical differences between the approaches in terms of rates of aortoenteric fistula recurrence. The difference in survival between the two types of approach reduced progressively over the years, secondary to a higher infection rate associated with the endovascular approach, although survival nevertheless remained higher in the endovascular group.^12^

Endovascular repair is a treatment option for aortoenteric fistulae, especially in patients with comorbidities and serious clinical status. Since it is conducted on an emergency basis, there are techniques that can be used to adapt devices available at the time for use in the endovascular repair procedure. There are several studies describing cases of intraoperative modification of aortic endoprostheses, whether by resection of a segment or by construction of fenestrations.^13^^,^^14^

Performing these modifications at the time of repair is particularly relevant in cases with complex anatomy, since custom-made devices take weeks to be produced.^14^ Considering the “on label” anatomic criteria for devices designed for aortic aneurysm repair, some authors claim that less than half of patients with pararenal and thoracoabdominal aneurysms meet the criteria for repair with “off-the-shelf” endografts, so tailoring devices to patients has a role to play in selected cases.^14^^,^^15^

Sweet et al.^16^ showed that, over the short term, physician-modified endografts used to treat thoracoabdominal aneurysms achieved results comparable to manufactured devices and could be used safely and effectively in patients with clinical conditions that were unfavorable for conventional surgical procedures.

The greatest concern in relation to customization of these devices is their durability, since there is an insufficient volume of studies to guarantee the durability and efficacy of modified devices over the long term. The same concerns were also present when endovascular aortic aneurysm repair was in its infancy. However, while there is no large-scale evidence, some publications support the long-term safety of modified devices.

Starnes et al.^17^ reported 12-month follow-up results from 59 patients who underwent juxtarenal aortic aneurysms using physician-modified devices. During the period studied, there were just two type III endoleaks, one type IB endoleak, and no type IA endoleaks.

In a more recent publication, the same group reported the results of 7-year follow-up of a juxtarenal aneurysm repair. This report also relates to physician-modified endoprostheses. Over 5 years, serial tomographic controls did not show structural failures, migration, or endoleaks and, on the contrary, they actually showed aortic remodeling with reduction in aneurysm sac diameters. In the seventh year of follow-up, the patient died from causes unrelated to aortic disease. A post-mortem study demonstrated that just one strut of the stent graft had fractured, without causing migration of the device, supporting the long-term efficacy and safety of physician-modified devices.^18^

There is no doubt that tailor-made and “off the shelf” endografts should be preferred, but the skills needed to modify devices should be part of surgeons’ therapeutic arsenals, primarily to cope with emergencies and for cases in which existing devices do not meet the anatomic demands of the patient. Furthermore, one should not ignore the idiosyncrasies of medicine in Brazil, which is a setting in which not all patients have unrestricted access to endovascular devices and, in such situations, the ability to modify endovascular devices can make a significant difference to patient prognosis.
