# Supplementary material for: Intraoperative endoprosthesis customization for repair of an aortoenteric fistula in an emergency context: a case report
Source: J Vasc Bras. 2021 Aug 2;20:e20200179. doi: 10.1590/1677-5449.200179 (PMC8336980; doi:10.1590/1677-5449.200179)
Supplement: Translation

# Translation: Customização intraoperatória de endoprótese para correção de fístula aortoentérica em contexto de urgência: um relato de caso

**Authors:** Hugo Back Carrijo, Josué Rafael Ferreira Cunha, Carlos André Schuler, Marcos Aurélio Perciano Borges

**Affiliations:** 1 Hospital de Base do Distrito Federal – HBDF, Brasília, DF, Brasil.; 2 Instituto de Cardiologia do Distrito Federal – ICDF, Brasília, DF, Brasil.

**Keywords:** fístula aortoentérica, aneurisma de aorta, procedimentos endovasculares, endopróteses modificadas

## Abstract

A fístula aortoentérica é uma grave condição clínica, e seu manejo permanece sendo um grande desafio técnico aos cirurgiões. A abordagem por cirurgia convencional nesses casos está relacionada a altos índices de morbimortalidade. A cirurgia endovascular apresenta-se como uma ótima alternativa nesses casos; contudo, por não se tratar de aorta nativa, a anatomia pode não ser compatível com os dispositivos endovasculares comercialmente disponíveis, fazendo-se necessário, em casos de urgência, a utilização de dispositivos modificados pelo cirurgião. O caso relatado reporta uma fístula aortoentérica secundária, tratada em situação de urgência por técnica endovascular com dispositivo modificado.

## INTRODUÇÃO

A fístula aortoentérica é uma complicação rara, mas que consiste em uma grave ameaça à vida. Em que pese ao seu longevo primeiro relato, por Ashley Cooper, datar de 1818^1^, ainda hoje representa um grande desafio terapêutico.

São classificadas como primárias e secundárias, sendo as primárias bastante incomuns, advindas de aneurismas volumosos, que causam a erosão do trato entérico adjacente, e as secundárias mais comuns, usualmente oriundas de enxertos aórticos com prótese, com prevalência entre 0,3 a 1,6% dos casos^2^. O quadro clínico inclui dor abdominal, hemorragia gastrointestinal e presença de massa abdominal pulsátil. Contudo, o diagnóstico costuma ser desafiador, visto que essa tríade clássica está presente em apenas 23% dos pacientes^3^^,^^4^.

O diagnóstico normalmente é feito por angiotomografia, método que apresenta ampla vantagem quando comparado a outros exames de imagem, visto ser exame de alta resolução, fácil acesso, pouco invasivo e com baixo tempo de aquisição. A cirurgia convencional para a correção da fístula aortoentérica secundária é tradicionalmente baseada na confecção de bypass extra-anatômico, ligadura da aorta e remoção do enxerto previamente interposto. No entanto, essa abordagem está associada a alta morbimortalidade: 25 a 90% de mortalidade operatória e 5 a 25% de amputação de membros inferiores^5^^,^^6^. Nesse contexto, a abordagem endovascular surge como uma opção terapêutica de grande valia para a correção de fístula aortoentérica, visto a gravidade desses pacientes e a morbidade do procedimento via cirurgia convencional.

No entanto, há de se levar em conta a anatomia complexa que muitos desses pacientes apresentam, em que as endopróteses disponíveis no momento da abordagem não se adequam aos critérios anatômicos do paciente. Dessa forma, modificações nas endopróteses são realizadas e descritas, com sucesso, por alguns autores, seja com ressecção de um segmento ou confecção de fenestrações. Essa abordagem é habitualmente reservada aos casos de urgência, para viabilizar a correção endovascular^7^^-^9.

O protocolo foi aprovado pelo Comitê de Ética de nossa instituição (parecer número 4.748.984).

## RELATO DE CASO

Um paciente do sexo masculino, de 66 anos, iniciou quadro de hemorragia digestiva alta, com dois episódios de hematêmese há dois dias da admissão hospitalar, mantendo vários episódios de melena desde então. Submetido, há 15 anos, à correção aberta por derivação aorto-femoral direito e ilíaca externa esquerda com prótese de Dacron, apresentava também como antecedentes hipertensão arterial sistêmica e doença pulmonar obstrutiva crônica.

Foi realizada angiotomografia de abdome que visualizou dilatação sacular na aorta abdominal infrarrenal em região proximal do bypass, fazendo interface com a quarta porção do duodeno, medindo 51x41 mm, sugestivo de pseudoaneurisma de anastomose proximal do enxerto, associado à fístula aortoentérica (Figura 1 e 2).

**Figura 1:** Angiotomografia de abdome em corte sagital demonstrando fistula aortoentérica em região da anastomose proximal de bypass aórtico prévio.

**Figura 2:** Angiotomografia de abdome em corte axial demonstrando fístula aortoentérica em região proximal da anastomose proximal de bypass aórtico prévio.

Por se tratar de fístula aortoentérica secundária, associada à prévia abordagem abdominal em paciente em grave estado clínico, optou-se por abordagem endovascular da lesão. Contudo, ao se realizar a reconstrução da angiotomografia e tomadas as medidas para planejamento do reparo endovascular, constatou-se que os parâmetros anatômicos eram inadequados, por dois motivos principais:

1. Diâmetro do colo proximal de 36 mm, impossibilitando a utilização de endopróteses bifurcadas. Vale lembrar que os maiores diâmetros disponíveis são da ordem de 36 mm;
2. Distância entre a artéria renal mais baixa e bifurcação da prótese de Dácron, utilizada no bypass aorto-femoral direito e ilíaca externa esquerda, de 89 mm, impossibilitando o uso de endoprótese de aorta torácica, visto que os dispositivos mais curtos apresentam 100 mm de extensão.

Sob essas condições anatômicas, portanto, não há dispositivo endovascular comercialmente disponível para empregar prontamente.

Dada a urgência e gravidade do caso, além da impossibilidade de customização de dispositivo endovascular, optou-se pela modificação intraoperatória da endoprótese torácica, Valiant Captivia Medtronic 40x167 mm (Medtronic, Minnesota, EUA).

Em primeiro lugar, foi realizada a liberação do dispositivo em mesa cirúrgica auxiliar “backtable” (Figura 3). Posteriormente, foi liberado o primeiro estágio da endoprótese Valiant Captivia Medtronic, mantendo fixo o seu anel aberto proximal. Após isso, foram realizadas as medidas do dispositivo e ressecção da extensão distal excedente com lâmina de bisturi (Figura 4).

**Figura 3:** Liberação da endoprótese Valiant Captivia Medtronic (Medtronic, Minnesota, EUA).

**Figura 4:** Ressecção de segmento distal da endoprótese Valiant Captivia Medtronic (Medtronic, Minnesota, EUA).

Em seguida, foi realizado o rencapamento da endoprótese, com auxílio de fita cardíaca, de forma lenta e progressiva, até a total cobertura de toda a extensão do dispositivo pelo sistema de entrega e liberação (Figura 5). Para o procedimento, foi realizada dissecção da artéria femoral direita com passagem de introdutor 7F e passagem de introdutor 5F em femoral esquerda por punção ecoguiada. A endoprótese foi introduzida pela femoral comum direita sob fio-guia Lunderquist (Cook Group Inc., Indiana, EUA), posicionada em situação infrarrenal, recobrindo o pseudoaneurisma de anastomose proximal do bypass aórtico prévio. Não houve intercorrência para a liberação da endoprótese e o controle final angiográfico demonstrou exclusão completa do pseudoaneurisma e resolução da fístula aortoentérica (Figura 6).

**Figura 5:** Rencapamento da endoprótese Valiant Captivia Medtronic (Medtronic, Minnesota, EUA).

**Figura 6:** Angiografia final demonstrando exclusão completa do pseudoaneurisma e resolução da fístula aortoentérica.

O paciente apresentou boa evolução no pós-operatório, recebendo alta hospitalar após 72h de internação, com boa aceitação da dieta oral, sem intercorrências e sem novos episódios de sangramentos.

## DISCUSSÃO

As fístulas aortoentéricas são uma das complicações mais graves do aneurisma de aorta e costumam apresentar altas taxas mortalidade pós-operatória^10^. A correção aberta está associada a grande morbidade cirúrgica, tendo em vista a dificuldade técnica de abordagem em abdome previamente operado e o risco de sangramento e contaminação significativo, associado à baixa patência da revascularização extra-anatômica^11^.

Uma revisão que analisou 216 artigos com um total de 823 pacientes com fistula aortoentérica demonstrou que a mortalidade intra-hospitalar na abordagem endovascular é menor que na abordagem aberta (7,1% contra 33,9%, respectivamente), sem diferenças estatísticas nas taxas de recorrência de fistula aortoentérica entre as abordagens. O benefício de sobrevida entre os tipos de abordagem diminuiu progressivamente ao longo dos anos, secundário a maior taxa de infecção na abordagem endovascular, porém mantendo, ainda assim, maior sobrevida nesse grupo^12^.

A correção endovascular é uma opção para tratamento das fistulas aortoentéricas, especialmente em pacientes com comorbidades e quadro clínico grave. Como o contexto em que é realizado é de urgência, há artifícios que podem ser utilizados para adequação do material disponível no momento para a correção endovascular. Há diversos estudos relatando casos de modificação intraoperatória de endopróteses aórticas, seja com ressecção de um segmento ou mesmo confecção de fenestras^13^^,^^14^.

A realização dessas modificações no momento da correção ganha relevância especialmente em casos de anatomia complexa, haja vista que dispositivos personalizados requerem semanas para serem produzidos^14^. Levando-se em conta os critérios anatômicos “on label” dos dispositivos para correção dos aneurismas de aorta, alguns autores discorrem que menos da metade dos pacientes com aneurismas pararrenais e toracoabdominais preenchem os critérios para correção com as endopróteses “off the shelf”, fazendo com que a personalização dos dispositivos tenha seu papel em casos selecionados^14^^,^^15^.

Sweet et al.^16^ demonstraram que as próteses modificadas pelo cirurgião para tratamento de aneurismas toracoabdominais apresentaram, em curto prazo, resultados comparáveis a dispositivos manufaturados, podendo ser utilizadas de forma segura e eficaz em pacientes com condições clínicas desfavoráveis para o procedimento cirúrgico convencional.

A maior preocupação em relação à customização desses dispositivos é quanto à durabilidade, visto a carência de volume de estudos que certifiquem a durabilidade e a eficácia desses dispositivos modificados a longo prazo. Essa preocupação também estava presente no início da terapia endovascular dos aneurismas de aorta. Contudo, apesar de não se contar com evidência em larga escala, algumas publicações ratificam a segurança dos dispositivos modificados ao longo do tempo.

Starnes et al.^17^ realizaram o seguimento por 12 meses de 59 pacientes submetidos à correção de aneurismas justarrenais de aorta, com dispositivos modificados por cirurgiões. Nesse período, foram identificados apenas dois endoleaks tipo III, um endoleak tipo IB e nenhum endoleak tipo IA.

Em recente publicação, os mesmos autores relataram o seguimento de 7 anos de correção de aneurisma justarrenal. No mesmo sentido, o relato observa endopróteses modificadas por cirurgião. Durante 5 anos, os sucessivos controles tomográficos não demostraram falhas estruturais, migração ou endoleaks, constatando-se, pelo contrário, um remodelamento aórtico com redução de diâmetro do saco aneurismático. No sétimo ano de seguimento, o paciente veio a falecer de causas não relacionadas a doença da aorta. O estudo post-mortem demonstrou a fratura de apenas uma haste de stent de endoprótese, sem causar migração do dispositivo, ratificando a efetividade e segurança a longo prazo de dispositivos modificados por cirurgiões^18^.

Não há dúvidas de que as endopróteses customizadas, ou “off the shelf”, devam ser priorizadas, mas a expertise em modificar os dispositivos deve estar presente no arsenal terapêutico dos cirurgiões, principalmente nas urgências e em casos em que os dispositivos não atendem as exigências anatômicas do paciente. Também não se pode esquecer das peculiaridades da medicina brasileira, com um cenário no qual nem todos os pacientes têm acesso irrestrito ao aparato endovascular, e, mais uma vez, nessas situações, a habilidade em modificar os dispositivos endovasculares pode representar uma significativa diferença no prognóstico dos pacientes.
